# Deoxynivalenol and Its Metabolites: Contamination, Metabolism, and Toxicity

**DOI:** 10.3390/toxins17110555

**Published:** 2025-11-11

**Authors:** Yukai Lin, Ruibiao Wang, Suxian Liu, Hanqing Zhao, Bo Wen, Songbiao Chen, Rongxian Guo, Lei Wang, Xiaojing Xia, Yanzhao Xu, Ke Ding

**Affiliations:** 1 College of Animal Science and Veterinary Medicine, Henan Institute of Science and Technology, Xinxiang 453003, China; 2 Laboratory of Functional Microbiology and Animal Health, Henan University of Science and Technology, Luoyang 471023, China; 3 Luoyang Key Laboratory of Live Carrier Biomaterial and Animal Disease Prevention and Control, Henan University of Science and Technology, Luoyang 471003, China; 4 The Key Lab of Animal Disease and Public Health, Henan University of Science and Technology, Luoyang 471023, China

**Keywords:** deoxynivalenol, feed contamination, metabolic pathways, toxicological effects

## Abstract

Deoxynivalenol (DON), a toxic secondary metabolite produced by *Fusarium graminearum* in infected cereal crops, has emerged as a major global contaminant in food and feed due to its stable physicochemical properties and resistance to degradation during conventional processing. This contamination poses a serious threat to livestock production and animal health. This review provides a comprehensive overview of the current status of DON contamination, its transmission through the food chain, metabolic pathways in animals, and the comparative toxicity of its metabolites. Furthermore, we analyze DON-induced toxic effects, including acute toxicity, cytotoxicity, immunotoxicity, neurotoxicity, gastrointestinal toxicity, and hepatotoxicity. By integrating domestic and international regulatory thresholds with current mitigation strategies, we highlight future research directions focusing on biodegradation technologies and genetic regulation approaches to alleviate DON contamination in livestock feeds. Advancing efficient DON-degradation strategies could open new avenues for sustainable feed management and mycotoxin detoxification technologies.

## 1. Introduction

Mycotoxins are toxic secondary metabolites produced by filamentous fungi that frequently contaminate grains, feed, and food commodities, thereby posing a significant threat to global agricultural security and human health [1]. Among them, deoxynivalenol (DON), a representative type B trichothecene mycotoxin, has garnered substantial attention in food safety and livestock production research due to its high prevalence and potent biological toxicity. The primary DON-producing species, *Fusarium graminearum* and *Fusarium culmorum*, synthesize and accumulate this toxin in wheat, maize, barley, and other crops under favorable temperature and humidity conditions [2]. Owing to its stable physicochemical properties (Table 1), DON resists degradation through conventional food-processing methods, facilitating its persistence and transmission across the feed supply chain to livestock and humans [3,4].

The deleterious effects of DON in animals are mediated through multifaceted mechanisms, including acute toxicity, growth retardation, disruption of the intestinal barrier, immunosuppression, and hepatotoxicity [12]. Studies indicate that swine exhibit markedly higher sensitivity to DON compared to poultry and ruminants. Chronic exposure to DON can lead to a reduction in feed conversion efficiency by 15–30%, resulting in annual economic losses exceeding USD 1 billion in the global livestock industry due to DON contamination [13,14]. Despite the establishment of regulatory limits for DON in animal feed across numerous countries, the global contamination rate of cereal remains alarmingly high.

Recent research has underscored concerns regarding the potential for in vivo reactivation of DON metabolites. While certain metabolites demonstrate reduced toxicity, DON-3-glucoside (D3G) is susceptible to enzymatic cleavage by gut microbiota, resulting in the release of the parent toxin and an increase in systemic toxicity. Additionally, the immunomodulatory effects of DON—ranging from immunosuppression to hyperactivation—and its intricate interactions with the gut microbiota are not yet fully elucidated, impeding the development of targeted mitigation strategies. This review provides a examination of the physicochemical properties and prevalence of DON and its metabolites, their metabolic pathways in animals, and the mechanistic associations between DON exposure and adverse effects on animal health.

## 2. Contamination in Food and Feed

### 2.1. Regulatory Maximum Limits for DON in Food and Feed

DON is among the most prevalent mycotoxins globally, with widespread contamination in cereals and animal feed, posing a significant threat to global food security and sustainable livestock production [15]. Regions with intensive grain production, such as China, Japan, Canada, and the Americas, are at a heightened risk of DON contamination. Grains compromised by DON not only suffer significant reductions in nutritional quality but also pose serious health risks to humans and animals, either through direct consumption or through bioaccumulation within the food chain [16]. To address the hazards associated with DON, international regulatory bodies have established stringent regulatory maximum limits for DON in both feed and food (shown in Table 2). Nevertheless, there remain inconsistencies in these standards across different countries and regions, which are influenced by variations in agricultural practices, dietary habits, and risk assessment methodologies. For example, China enforces a DON limit of 1000 µg/kg in feed for susceptible species such as pigs and calves, while the European Union imposes a more stringent limit of 200 µg/kg for cereal products intended for infants. It is noteworthy that higher tolerance limits for DON are typically applied to ruminant feeds, due to the partial detoxification capacity of the rumen microbiota through microbial biotransformation.

### 2.2. DON Contamination

DON is predominantly detected in animal feeds and cereals, including oats, maize, wheat, and barley, and is prevalent in regions such as East Asia, Northern and Central Europe, Central America, North America, and South Africa [22]. The highest levels of DON have been reported in wheat samples from specific areas in China [23]. In a Shanghai-based study, DON was detected in 85.8% of wheat flour samples [24]. In a survey conducted in Hebei Province from 2011 to 2013, DON was detected in 91.5% of wheat samples, with a median concentration of 154 µg/kg and a maximum level reaching 1130 µg/kg [25]. Similarly, in Shandong Province, the contamination rate of DON in wheat flour was as high as 97.2% [26]. Beyond China, significant DON contamination has also been reported in other regions. For instance, in Europe, contamination rates approached 100% in German silage maize and Polish complete feed [27,28]. In the United States, DON was detected in 94% of swine feed samples and in 88.2% of corn silage samples, with certain cases exceeding 5000 µg/kg [29,30]. Additionally, in Brazil, DON was detected in 77.9% of wheat flour samples, with the highest concentration reported at 2794.63 µg/kg [31]. The occurrence of DON contamination exhibits notable regional and ingredient specificity (Table 3). These high contamination rates may be attributed for *Fusarium* growth in temperate climates. Contamination rates were generally higher in silage and piglet feed compared to processed grains microbial biotransformation in the rumen, indicating that feed ingredients are particularly vulnerable to secondary contamination during storage and fermentation. The global economic impact of DON contamination on livestock is substantial, with Asia and Europe bearing a relatively high burden.

### 2.3. Transmission in the Food Chain

DON is predominantly synthesized by *Fusarium* spp., with its biosynthesis being markedly influenced by environmental conditions. Research indicates that *Fusarium* spp. can readily infect cereal crops such as wheat, maize, and barley under optimal temperature and humidity conditions, continuing to produce DON throughout their growth phase [36,37]. The distribution of DON within plant tissues is notably heterogeneous. In wheat, DON is primarily concentrated in the bran layer, leading to significantly higher concentrations in wholemeal flour compared to refined flour [38]. The data show that whole grain products generally contain higher DON concentrations than refined flour products, which is consistent with the preferential accumulation of DON in the bran layer. In maize, DON levels are frequently higher in the stem than in the ear, and variations in environmental conditions across different years can result in distinct differences in DON accumulation by altering the pattern of *Fusarium graminearum* infestation. This heterogeneous distribution has direct implications for the safety risk assessment of silage and processing by-products [39].

Furthermore, DON enters the livestock production chain through the consumption of contaminated crops used as feed ingredients. DON is partially absorbed by the digestive tract of animals, after which its parent compound and metabolites are distributed to tissues such as the liver and kidneys via the bloodstream and subsequently excreted through urine and feces. Nonetheless, trace amounts of DON and its metabolites may persist in muscle tissue, liver, and dairy products, ultimately entering the human food chain through consumption of animal-derived products such as meat, eggs, and milk [40,41]. The transfer of DON within the food chain is further complicated by the effects of food processing. Due to its high chemical stability, DON remains present throughout grain harvest, storage, and transport. It can persist into subsequent food-processing stages. Conventional processing techniques result in only minimal reductions in DON levels, and concentrations in processing by-products may be elevated due to water loss. Epidemiological studies indicate that exposure to DON can result in acute toxicity, while chronic low-dose exposure is associated with immunosuppression, intestinal barrier damage, and neurological dysfunction, thereby posing a significant risk to both human and animal health [42,43].

## 3. Biotransformation and Metabolites of DON

### 3.1. Secondary Metabolites of DON

**Figure 1:**
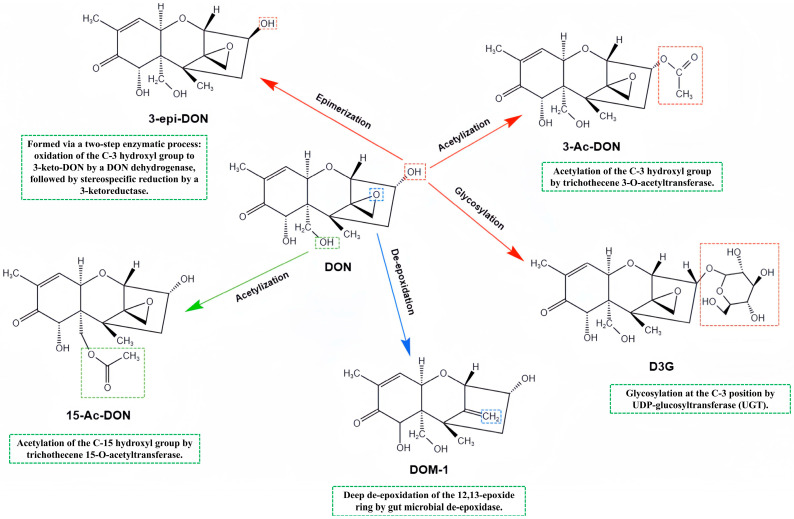
Structures and enzymatic formation pathways of DON and its metabolites.

DON undergoes metabolism in organisms through various pathways, resulting in the formation of secondary metabolites with diverse structures. The primary secondary metabolites of DON currently identified include 3-Acetyl-DON, 15-Acetyl-DON, DON-3-glucoside (D3G), de-epoxylated-DON (DOM-1), and 3-epi-DON, among others (Figure 1) [44].

Both 3-acetyl-DON and 15-acetyl-DON, acetylated derivatives of DON commonly found in contaminated food and feed, can be deacetylated by intestinal microorganisms to revert to DON, thereby exhibiting acute and chronic toxicities similar to the parent compound [45]. D3G, a detoxification product resulting from the glycosylation of DON in plants, possesses low inherent toxicity. However, it may be hydrolyzed by intestinal flora to release free DON, potentially increasing the risk of toxicity [46]. DOM-1 is generated through microbial or enzyme-catalyzed de-epoxidation of DON and displays markedly reduced toxicity due to the loss of the epoxy group, which lowers its affinity for the ribosome and consequently its inhibition of protein synthesis [47]. Furthermore, 3-epi-DON is produced through isomerization, resulting in an altered stereochemical conformation that diminishes its interactions with biomolecules, thereby exhibiting reduced cytotoxicity [48].

The formation mechanisms of DON secondary metabolites are intricate, involving multiple pathways such as microbial transformation, plant detoxification, and animal metabolism. Microorganisms modify the structure of DON through enzymatic processes including acetylation, deacetylation, de-epoxidation, and isomerization, leading to the production of corresponding metabolites [49,50,51]. In plants, DON is conjugated with glucose by glycosyltransferases to form D3G, thereby reducing its toxicity. In animals, liver metabolizing enzymes facilitate de-epoxidation, glucuronidation, and sulfonation processes, generating various metabolites and enhancing the excretion of DON.

Compared to DON, DOM-1 and 3-epi-DON exhibit lower toxicity and have reduced inhibitory, induction of oxidative stress, and apoptosis-promoting effects on cell proliferation [52,53]. From a molecular perspective, DON robustly activates the MAPK signaling pathway, leading to inflammatory responses and apoptosis. In contrast, this pathway’s activation is markedly reduced by the presence of DOM-1, indicating that the toxic effects of DON are intricately linked to structural modifications [54]. These observations underscore the necessity of integrating toxicity assessments of secondary metabolites with their chemical characterization and metabolic dynamics to comprehensively elucidate their potential impacts on organisms.

### 3.2. Metabolism of DON in the Organism

#### 3.2.1. Humans

Human exposure to DON occurs primarily through the consumption of contaminated cereals and cereal-based products [1]. Following ingestion, DON is efficiently absorbed in the gastrointestinal tract [55]. The major metabolic pathway involves glucuronidation, catalyzed by uridine diphosphate-glucuronosyltransferases (UGTs) in the liver and other tissues, leading to the formation of DON-glucuronide conjugates, such as DON-15-glucuronide and DON-3-glucuronide [56]. Furthermore, the human intestinal microbiota hydrolyzes the plant conjugate DON-3-glucoside (DON-3G) to release free DON, and in certain individuals, further transforms it into the de-epoxy metabolite DOM-1, which exhibits lower toxicity [57]. DON and its metabolites are rapidly excreted, predominantly via the kidneys, with approximately 64% of ingested DON and 58% of DON-3G recovered in urine within 24 h post-administration [58]. Consequently, urinary DON and its glucuronide conjugates, particularly DON-15-glucuronide, serve as robust biomarkers for assessing human exposure. Numerous biomonitoring studies conducted globally in regions such as China, Belgium, Italy, and the United Kingdom have employed these biomarkers for exposure assessment, revealing that certain population subgroups, especially children, may experience exposure levels exceeding the established tolerable daily intake (TDI) [59,60,61,62,63,64].

#### 3.2.2. Monogastric Animals

Swine are highly sensitive to DON. The porcine gastrointestinal tract rapidly and almost completely absorbs this mycotoxin, resulting in high systemic bioavailability. Following oral intake, DON is primarily absorbed in the proximal small intestine. Plasma concentrations peak approximately 4.1 h post-feeding, with an elimination half-life (t_1_/_2_) of about 5.8 h [65]. Inside the body, DON is predominantly metabolized in the liver and kidneys. This process, catalyzed by UGTs, leads to the formation of DON-glucuronide conjugates (DON-GlcA). DON-GlcA represents the major form in plasma and serves as a suitable biomarker for assessing DON exposure [66]. Furthermore, DON and its metabolite de-epoxy-DON can be detected in serum, liver, bile, and urine. Their concentrations demonstrate a significant linear increase corresponding to rising dietary DON levels [67]. Renal excretion is the primary elimination route for DON in swine. Up to 43.2% of the ingested dose can be excreted as the parent toxin in urine, while fecal excretion is considerably lower (approximately 3.0%) [68]. The strong correlation between DON intake and its concentrations in both serum and urine confirms these matrices as reliable indicators for monitoring DON exposure in swine [69].

Compared with swine, poultry exhibit lower sensitivity to DON, which is attributed to their distinct metabolic pathways. The intestinal absorption of DON in poultry is limited, with an oral bioavailability of only 19.3%. In broilers, DON is rarely detected in its parent form in plasma; instead, it is rapidly and extensively converted to its major metabolite, DON-3-sulfate (DON-3S). Studies have shown that after 42 days of being fed diets containing 5 mg/kg or 15 mg/kg DON, DON-3S was detectable in the plasma and excreta of broilers. However, hepatic deposition was observed only in the high-dose group (15 mg/kg). Therefore, DON-3S can serve as a specific biomarker for DON exposure in various biological matrices (plasma, excreta, liver) in poultry [70]. Apart from DON-3S, excreta contained only minimal amounts of the parent DON, and no de-epoxidation metabolite (DOM-1) was detected. Poultry excrete DON rapidly, primarily via feces, with a reported biorecovery (based on DON-3S and DON) of 74–106% in broilers. The conversion of DON to DON-3S is considered a detoxification mechanism, as in vitro studies indicate that the toxicity of DON-3S to ribosomes is nearly 2000 times lower than that of DON [71]. The relative tolerance of poultry to DON is likely due to their efficient conversion of DON into the less toxic DON-3S and its rapid elimination. Chronic DON exposure in broilers may induce intestinal adaptive changes, such as increased jejunal length and reduced villus height; however, nutrient retention remains unaffected, suggesting the presence of physiological adaptation mechanisms [72].

#### 3.2.3. Ruminants

Ruminants exhibit relatively low sensitivity to DON, primarily due to the potent degradation capacity of rumen microbiota. Under normal rumen function, rumen microbes efficiently convert ingested DON into the nearly non-toxic de-epoxy metabolite DOM-1, with conversion rates reaching 81–99%. Consequently, DOM-1 is the primary compound detected in the blood and milk of ruminants, while the systemic bioavailability of DON itself is very low (e.g., only 5.9–9.9% in sheep) [73]. However, this detoxification capability is highly dependent on a healthy rumen environment. In vitro studies indicate that when rumen pH drops to 5.8 (simulating subacute ruminal acidosis, SARA), DON degradation is significantly inhibited. This is particularly evident in rumen fluid from non-lactating cattle with lower microbial activity, where the degradation rate can decrease to approximately 40% [74]. High-starch diets predispose the rumen to pH depression, which not only reduces DOM-1 production but also increases the absorption of unmetabolized DON. These changes are associated with decreased total ruminal volatile fatty acid concentrations and elevated serum inflammatory markers (e.g., TNF-α) in dairy cows [75]. In summary, maintaining healthy rumen function is crucial for protecting ruminants against the adverse effects of DON. Under conditions of SARA or impaired rumen microbial activity, DON may escape degradation, leading to increased post-ruminal exposure and potential health risks.

## 4. Effects of DON on the Health of the Organism

### 4.1. Toxic Effects of DON

**Figure 2:**
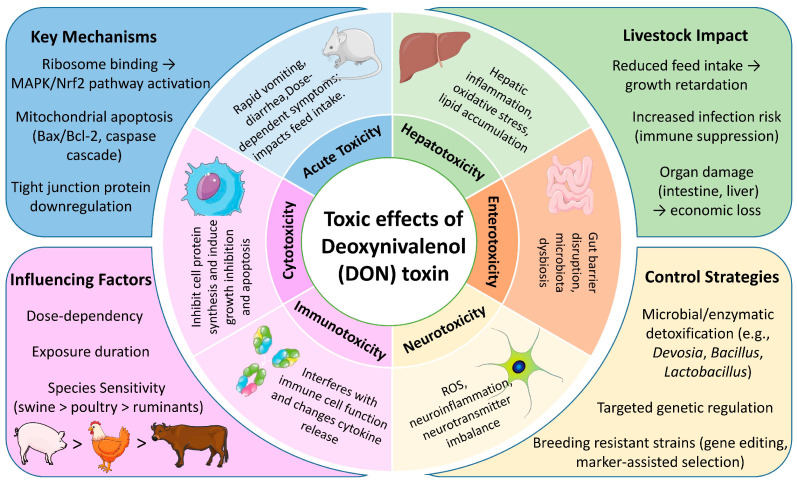
Toxic mechanisms, impacts, and integrated control strategies of DON.

The health impacts of DON on animals are multifaceted. By binding to ribosomes, it can trigger cellular stress and apoptosis, and disrupt intestinal tight junctions. This leads to a series of toxic effects including cytotoxicity, immunosuppression, intestinal barrier damage, and hepatotoxicity, ultimately resulting in growth retardation and economic losses in livestock and poultry (Figure 2) [76].

#### 4.1.1. Acute Toxicity

The acute toxicity of DON is primarily manifested by rapid physiological responses following high-dose exposure in both humans and animals, inducing symptoms such as vomiting, diarrhea, dizziness, and disorders of the central nervous system, with severity influenced by species, sex, age, and exposure route [77,78]. In humans, acute exposure through contaminated food, particularly during cereal-related outbreak events, has been clinically associated with gastroenteritis characterized by vomiting, abdominal pain, and diarrhea [79]; findings from Chinese epidemiological studies further substantiate the emetic potential of DON in human populations [80]. This emetic property, which earns DON the common name “vomitoxin,” has been rigorously quantified in sensitive animal models such as the pig. It has been demonstrated in mouse studies that exposure to 1 and 2.5 mg/kg·bw of DON can induce acute anorexia and concurrently elevate plasma levels of the intestinal hormones CCK, PYY, GIP, and GLP-1 within 3 h [81]. Among livestock, pigs exhibit the highest sensitivity, achieving peak plasma levels within 30 min post-ingestion of contaminated feed, with vomiting occurring approximately 10 min thereafter [5,82]. Studies have reported the minimum emetic dose (MED) in pigs weighing 9–10 kg to be 0.05 mg/kg body weight (BW) via intraperitoneal injection and 0.1–0.2 mg/kg BW via oral gavage [83]. In contrast, ruminants demonstrate greater tolerance due to the de-epoxygenation process facilitated by rumen microorganisms, with poultry displaying intermediate sensitivity. The acute toxicity of DON has direct implications for livestock production, manifesting as reduced feed intake, growth inhibition, and organ damage, all of which collectively result in economic losses [84,85].

#### 4.1.2. Cytotoxicity

DON exerts its effects by binding to the 60S subunit of eukaryotic ribosomes, thereby inhibiting peptidyltransferase activity, disrupting protein synthesis, and activating the MAPK signaling pathway. This activation induces a “ribosomal stress response”, culminating in apoptosis [86]. In livestock, intestinal epithelial cells and immune cells are particularly susceptible to DON’s effects. Specifically, the viability of porcine small intestinal epithelial cells (IPEC-J2) is significantly compromised upon exposure to 1 μM DON, with cell membrane rupture and vacuolization observed at concentrations exceeding 8 μM [87]. Similarly, in human cells such as keratinocytes, DON exposure significantly decreases cell viability and induces oxidative stress, mitochondrial damage, and cell death through apoptosis and pyroptosis [88]. DON exhibits dose-dependent cytotoxicity, disrupting cell membrane integrity, inflicting DNA damage, altering cellular morphology, impairing protein synthesis, and ultimately inducing apoptosis. Additionally, DON contributes to the formation of mitotic-positive micronuclei, which can further exacerbate organ damage [89].

#### 4.1.3. Immunotoxicity

DON functions as a bi-directional modulator of the immune system, inducing nucleotoxic stress and inhibiting the synthesis of RNA, DNA, and proteins [90]. The immunotoxic effects of DON are modulated by various factors, including exposure dose, duration, and host physiology. These effects are characterized by an imbalance between humoral and cellular immunity, abnormal serum IgA levels, increased lymphocyte apoptosis, and disruptions in the expression of pro-inflammatory factors [5,91]. At the molecular level, DON activates the endoplasmic reticulum stress-related signaling pathway (ATF3/DDIT3) and mediates mitochondria-dependent apoptosis through the activation of the MAPK phosphorylation cascade as well as hematopoietic cell kinase (Hck), ultimately inhibiting the proliferation and differentiation of immune cells [92]. At low concentrations (<1 μM), DON enhances host defense against pathogens, as evidenced by the up-regulation of T-cell activating factors, enhancement of helper T-cell function, and increased intestinal IgA secretion [93]. When the concentration of DON exceeds 1 μM, its immunosuppressive effects became predominant, resulting in the dysregulation of interleukin-mediated immunoglobulin secretion. Additionally, DON inhibited the expression of inducible nitric oxide synthase (iNOS) in intestinal epithelial cells through the proteasomal pathway, leading to intestinal inflammation and an elevated risk of infection [94]. Furthermore, acetylated derivatives and bound-state metabolites of DON exhibit similar immunomodulatory activities. Notably, deoxynivalenol-3-glucoside (DOM) can specifically activate humoral immune responses due to its preserved ability to bind to lymphocyte surface receptors [93]. The immunotoxicity of 3-acetyldeoxynivalenol (3ADON) and 15-acetyldeoxynivalenol (15ADON) is significantly lower than that of DON. In contrast, deoxynivalenol-3-glucoside (D3G) exhibits negligible immunotoxicity, as it does not induce cytokine release nor trigger a nucleotoxic stress response [94].

#### 4.1.4. Neurotoxicity

The neurotoxic mechanism of DON is intricate, involving interactions across multiple pathways and resulting in multi-target damage. Research has demonstrated that DON can traverse the blood–brain barrier and exert direct effects on the central nervous system, altering its permeability and inducing dysfunction in both neuronal and glial cells. The toxicological effects of DON are primarily manifested through mitochondria-mediated apoptosis, oxidative stress, calcium signaling dysregulation, neuroinflammation, glial cell injury, and disruption of neurotransmitter systems. DON induces oxidative stress and neuronal apoptosis by activating the mitochondrial apoptotic pathway, characterized by an imbalance in Bax/Bcl-2, the release of cytochrome C, and the caspase-9/caspase-3 cascade, while also modulating the MAPK signaling pathway. Additionally, the activation of caspase-8 further amplifies the apoptotic effects via the mitochondrial pathway [95,96]. Regarding oxidative stress, exposure to DON markedly increased reactive oxygen species (ROS) levels, resulting in lipid peroxidation, protein carbonylation, and DNA damage. This exposure also precipitated a collapse in mitochondrial membrane potential and disrupted neurotransmitter metabolism by interfering with Ca^2+^ homeostasis, particularly affecting the Ca^2+^/CaM/CaMKII signaling pathway [97]. DON prompts microglia to secrete pro-inflammatory cytokines, such as TNF-α, and activates inflammatory responses in astrocytes, thereby intensifying central nervous system (CNS) inflammation [98]. Additionally, Drp-1 (Dynamin-related protein 1)-mediated mitochondrial hyperfission and the activation of autophagy further contribute to neuronal degenerative pathology [99]. Furthermore, DON disrupts the neurotransmitter system by significantly reducing levels of dopamine and gamma-aminobutyric acid while increasing concentrations of serotonin and norepinephrine. This neurotransmitter imbalance is closely linked to appetite suppression, gastrointestinal dysfunction, and behavioral abnormalities [42].

#### 4.1.5. Enterotoxicity

The intestinal tract serves as the primary target organ for DON in both humans and animals after consumption of contaminated food or feed, leading to morphological damage, impaired nutrient absorption, and compromised barrier function. Chronic exposure thus contributes to significant intestinal pathology. For example, in young rats given 10 mg/g DON for 28 days, jejunal villi exhibited shortening, fusion, and increased epithelial apoptosis [100]. Similarly, broilers and pigs fed 10 mg/kg DON for 8 weeks showed dose-dependent reductions in villus height, increased crypt depth, and decreased villus-to-crypt ratio, alongside marked downregulation of tight junction proteins, indicating impaired barrier integrity [101]. Notably, chronic exposure to DON at a human-relevant dose (10 μg/kg bw/day) in mice disrupts multiple aspects of intestinal homeostasis, encompassing microbiota composition, immune response, and epithelial integrity, which bridges the toxic effects observed in livestock to potential human health risks [102]. DON also markedly alters the gut microbiota. In weaned piglets, DON reduced Firmicutes abundance and increased Actinobacteria, indicating dysbiosis [103]. Weaned rabbits exposed to high DON levels showed decreased microbial diversity, further destabilizing the gut microenvironment [104]. Moreover, DON impairs epithelial repair by inhibiting proliferation and inducing apoptosis. Piglets chronically exposed to low-dose DON had fewer jejunal and ileal epithelial cells and a higher proportion of apoptotic cells, suggesting disrupted cellular homeostasis aggravates intestinal dysfunction [105,106]. In summary, evidence from diverse animal models, including those relevant to humans, demonstrates that DON-induced intestinal toxicity involves structural damage, barrier defects, microbial imbalance, and loss of cellular homeostasis, collectively endangering host health.

#### 4.1.6. Hepatotoxicity

As a central organ involved in metabolism, detoxification, and immunoregulation, the structural and functional integrity of the liver is vital for maintaining overall physiological homeostasis. Research has demonstrated that exposure to DON can significantly impact liver morphology and function, as evidenced by alterations in the ultrastructure of hepatocytes. In porcine hepatocytes treated with DON, numerous short, dilated vesicles were observed in both the rough and smooth endoplasmic reticulum, suggesting potential interference with intracellular protein synthesis and lipid metabolism [107]. In animal models, histopathological analyses of the livers of broilers and pigs fed with high doses of DON revealed inflammatory cell infiltration in liver tissue, accompanied by increased liver injury [101]. The deleterious effects of DON on the developing liver are notably pronounced, with pathological alterations such as the accumulation of lipid vesicles in hepatic tissue and a marked suppression of splenic immune function observed in the chick embryo exposure model [108]. At the molecular level, both in vitro and in vivo studies have elucidated that DON exacerbates hepatic injury by inducing inflammatory responses, activating oxidative stress pathways, and promoting apoptosis in hepatocytes. For instance, short-term exposure to DON significantly diminishes the viability of HepG2 cells and exerts direct toxic effects on primary hepatocytes and liver tissue [109]. Furthermore, animal studies have demonstrated that DON exposure disrupts hepatic redox homeostasis, as indicated by increased lipid peroxidation products and decreased activity of antioxidant enzymes, alongside the aberrant expression of pro-inflammatory factors [110,111]. These findings collectively elucidate the multi-faceted mechanisms of DON-induced hepatic toxicity, providing a critical foundation for assessing its health risks and developing targeted prevention and mitigation strategies.

### 4.2. Effects on Biochemical Indicators

DON significantly influences serum biochemical indices across a broad spectrum of animal species, exhibiting effects that are both consistent and species-specific. Serum biochemical markers are crucial for evaluating the homeostasis of the internal environment and organ functionality in animals, with aberrant changes often indicative of metabolic disturbances or tissue damage.

In pigs, DON exerts significant dose-dependent effects on biochemical indices. Research has demonstrated that prolonged consumption of low-dose DON (12 μg/kg body weight) by prepubertal sows led to reductions in blood alanine aminotransferase levels, erythrocyte counts, platelet, and leukocyte counts, thereby disrupting blood homeostasis [112]. Conversely, when healthy growing pigs were fed a high dose of DON (12 mg/kg), there was an increase in blood urea nitrogen, alkaline phosphatase, and alanine aminotransferase levels, alongside a decrease in serum L-valine, glycine, and other amino acid concentrations. This was accompanied by a reduction in antioxidant enzyme activities, suggesting that DON can induce metabolic disorders and oxidative stress [113]. Recent experimental studies on piglets have corroborated that diets contaminated with DON markedly decrease average daily weight gain and feed intake. These diets also suppress the activities of antioxidant enzymes, including serum immunoglobulin G, catalase, and superoxide dismutase, and downregulate the expression of anti-inflammatory cytokines such as IL-4 and IL-10. Conversely, there is an upregulation of pro-inflammatory cytokines, including TNF-α, interleukin-6, and interleukin-12 [114].

Parallel investigations in the poultry sector have demonstrated the dose-dependent toxicity of DON. In chicks administered a diet with 10 mg/kg DON until 35 days of age, there was a significant reduction in serum alanine aminotransferase (ALT) activity, alongside significant increases in cholesterol and triglyceride levels. These findings indicate disruptions in lipid metabolism and a suppression of the humoral immune response to viral vaccines [115]. Lower doses (5 mg/kg) of DON did not affect growth performance but reduced serum creatine kinase levels and altered small intestine morphology. In contrast, higher doses (15 mg/kg) not only reduced body weight gain and feed efficiency but also decreased serum cholesterol levels. Additionally, higher doses led to increased absolute and relative weights of the thymus and gasterocysts, along with a reduction in colon weights [116]. These findings indicate that DON exerts a dose-dependent effect on the biochemical indices of chickens, involving the comprehensive regulation of lipid metabolism, organ development, and immune function, with more pronounced toxic effects at higher doses.

In fish, the impact of DON on biochemical parameters varies considerably among species and exposure conditions. For instance, the intake of DON (1.15–6 mg/kg feed) by *Atlantic salmon* (*Salmo salar*) resulted in dose-dependent decreases in plasma total protein, albumin, cholesterol, and triglyceride levels, as well as a reduction in alkaline phosphatase activity [117]; in red tilapia (*Oreochromis niloticus* × *O. mossambicus*), total plasma protein and lipid indices did not show significant changes after ingestion of DON-containing diets (0.07–1.15 mg/kg), but focal necrosis and cytoplasmic vacuolization were observed in liver tissues [118]; rainbow trout (*Oncorhynchus mykiss*) showed significant reductions in plasma glucose, cholesterol, and ammonia levels, as well as a decrease in the mean hemoglobin content of erythrocytes (MCH), when exposed to DON (2 mg/kg feed) [119]. These differences are closely related to factors such as fish species sensitivity, toxin exposure concentration and experimental design.

Overall, DON exerts widespread effects on the serum biochemical indices of various species, including pigs, poultry, and fish, by disrupting metabolic enzyme activities, inducing oxidative stress, compromising intestinal barriers, and suppressing immune functions. The toxic effects of DON are closely associated with both the exposure dose and duration.

### 4.3. Reproductive Effects

DON poses a serious threat to the reproductive health of various mammals. In males, DON exposure can induce testicular toxicity, manifested as exfoliation of sperm cells, decreased sperm quality and concentration, and increased malformation rates [120]. The mechanism is primarily associated with DON-induced oxidative stress, which activates the assembly of the NLRP3-ASC-Caspase 1 inflammasome, leading to pyroptosis of Leydig cells. This results in testosterone deficiency and elevated serum levels of FSH and LH [121]. Moreover, DON disrupts the integrity of the blood-testis barrier (BTB) by downregulating the expression of junctional proteins such as Occludin, Connexin 43, and N-cadherin. These alterations, together with inflammatory responses and hormonal imbalance, ultimately interfere with spermatogenesis [122]. In females, DON exerts detrimental effects on ovarian morphology, causing degeneration of oocytes and granulosa cells, interstitial edema, and a reduction in the number of normal follicles at various developmental stages, thereby impairing folliculogenesis [123]. Such ovarian damage is accompanied by alterations in serum FSH levels and inflammatory responses, with susceptibility exhibiting age-specific differences between prepubertal and adult females [124]. During pregnancy, maternal DON exposure also adversely affects placental structure and immune function, characterized by downregulation of junctional proteins, elevated levels of inflammatory cytokines (IFN-γ, IL-6, IL-4), and reduced fetal survival rates [125]. In summary, current evidence indicates that DON-induced reproductive toxicity is mainly mediated by interconnected mechanisms including oxidative stress, inflammatory responses, apoptosis, and pyroptosis, highlighting the necessity for further risk assessment and the development of targeted intervention strategies.

## 5. Strategies for DON Contamination Control: Biodegradation and Genetic Regulation

Deoxynivalenol, a prevalent mycotoxin in grains and feed, seriously threatens livestock and human health. Conventional detoxification methods face challenges including limited efficacy, nutrient loss, and hazardous by-products. Biodegradation and genetic regulation have thus gained attention as promising alternatives due to their efficiency, specificity, and environmental safety. This section reviews microbial and enzymatic biodegradation approaches alongside genetic strategies such as gene editing and transgenic technology, offering a theoretical basis for integrated DON control.

### 5.1. Biodegradation Technology

Biodegradation technology primarily utilizes microorganisms and their specific enzymes to convert deoxynivalenol (DON) into less toxic or non-toxic metabolites, representing an effective strategy for in situ detoxification of DON in feed and food products. Numerous studies have demonstrated the capacity of various bacterial strains to efficiently degrade DON. For instance, Devosia insulae A16 has been shown to oxidize DON to 3-keto-DON under aerobic conditions, achieving a degradation rate of 88% within 48 h [126]. Strains such as Bacillus sp. HN117 and N22 are capable of degrading DON even at high concentrations (up to 500 mg/L), yielding less toxic metabolites including M-DOM, norDON E, and 9-hydroxymethyl DON lactone [3]. Furthermore, Lactobacillus rhamnosus MY-1 exhibited a high DON degradation rate of 93.34%, and in mouse models, it significantly improved growth performance and intestinal health [127]. Fungal species such as Aspergillus tubingensis NJA-1 achieved a 94.4% biotransformation rate of DON via hydrolysis [128]. In the case of yeast, Saccharomyces cerevisiae boulardii CNCM I-1079 was reported to counteract DON-induced activation of NF-κB and p38 MAPK inflammatory pathways, while also restoring lipid metabolism and antioxidant pathways [129]. At the enzymatic level, *Devosia mutans* 17-2-E-8 employs a two-enzyme system comprising DepA and DepB to catalyze the epimerization of DON to 3-epi-DON. In this process, DepB functions as an NADPH-dependent dehydrogenase, reducing 3-keto-DON to the final product, 3-epi-DON [130]. Additionally, the soil bacterium *Sphingomonas* sp. S3-4, along with the recombinant aldo-keto reductase AKR18A1, catalyzes the oxidation of DON to 3-oxo-DON, which is subsequently converted into the non-phytotoxic 3-epi-DON, facilitating DON degradation [131]. Collectively, these findings underscore the considerable potential of microbial and enzymatic approaches for the biodegradation of DON, highlighting their promising applications in enhancing food and feed safety.

### 5.2. Gene Regulation Technology

Beyond biodegradation methods, genetic regulation strategies have emerged as pivotal approaches for mitigating DON contamination, acting through direct targeting of mycotoxin biosynthetic pathways or enhancement of host resistance mechanisms. In *Fusarium graminearum*, key transcription factors Tri6 and Tri10 govern DON biosynthesis by regulating the expression of TRI genes, including TRI5. Concurrently, a long non-coding RNA (RNA5P) exerts cis-repressive effects on TRI5 expression and DON production [132]. Furthermore, the zinc-finger transcription factor FgSfp1 coordinates DON synthesis by modulating ribosome biogenesis and post-transcriptional RNA modification; its disruption results in a 95.4% reduction in DON production [133]. CRISPR-based functional genomics screening has identified the host gene MDH2 as a central mediator of the cytotoxicity of multiple *Fusarium* toxins, including DON. Knockout of MDH2 enhances cellular toxin tolerance [134]. In wheat, specific SNPs in the UDP-glucosyltransferase genes TaUGT-2B and TaUGT-3B have been shown to confer resistance to Fusarium head blight and reduce DON accumulation by modulating their expression during early pathogen infection [135]. Collectively, these genetic and molecular insights not only deepen our understanding of DON toxicity and biosynthesis but also provide critical targets and practical strategies for developing novel control measures through gene editing, marker-assisted breeding, and transgenic technologies.

## 6. Conclusions and Outlook

In light of the global prevalence and persistent threat of deoxynivalenol (DON) contamination in grains and feed, this review synthesizes current understanding of its contamination status, species-specific metabolic pathways, and multifaceted toxicity mechanisms—including immunotoxicity, neurotoxicity, enterotoxicity, hepatotoxicity, and reproductive impairment—while also evaluating existing mitigation strategies such as microbial detoxification, enzymatic degradation, and genetic regulation. Looking forward, future efforts should prioritize elucidating threshold effects of DON during critical physiological stages, refining real-time detection and differentiated regulatory standards, and advancing integrated solutions combining biodegradation agents, resistant crop varieties, and precision feeding management. By bridging mechanistic insights with practical interventions, we can ultimately mitigate DON’s impact on animal health and foster the sustainable development of livestock production systems.

## Figures and Tables

**Table 1:** Physicochemical properties of deoxynivalenol.

Categories	Information	References
IUPAC Naming	12,13-epoxy-3α,7α,15-trihydroxytrichothec-9-en-8-one	[5]
Physical state	Colorless fine needles	
Molecular formula	C_15_H_20_O_6_	
Solubility	It is soluble in polar organic solvents (such as methanol, ethanol, chloroform, acetonitrile, and ethyl acetate) and water, but insoluble in n-hexane, butanol, and petroleum ether.	
Flash point	206.9 ± 2.5 °C	
Melting point	151–153 °C	
Molecular mass	296.32 g/mol	
Boiling point	500–550 °C	
Thermal stability in food processing	Under conditions of baking at 210 °C, frying at 140 °C, or boiling, the degradation rate of DON is only approximately 50%.	[6,7,8]
Stability in response to temperature, duration, and pH	1. At pH 4, the chemical structure of DON remained intact after heating at 100 °C and 120 °C for 60 min, with only minor degradation occurring at 170 °C for 60 min. 2. At pH 7, it remained highly stable at 100 °C and 120 °C for 60 min, while partial degradation was observed at 170 °C for 15 min. 3. At pH 10, partial degradation occurred at 100 °C after 60 min; complete destruction was achieved after exposure to 120 °C for 30 min or 170 °C for 15 min.	[9]
Long-term storage stability	DON exhibits extremely high stability in cereals and their products and can retain its toxicity for an extended period.	[10,11]

**Table 2:** Regulatory maximum limits for DON in food and feed in selected different countries and regions.

Country/Region	Commodity		Maximum Limits (μg/kg)	References
China	Food	Corn, cornmeal (residue, flakes)	1000	[17,18]
		Barley, wheat, muesli, wheat flour	1000	
	Feed	Calves, Lambs, Lactation Concentrate Supplements	1000	
		Pig compound feed	1000	
		Plant-based feed ingredients	5000	
USA	Food	Finished wheat products (flour, bread)	1000	[19]
	Feed	Grains and grain byproducts for swine	5000	
		Grains and grain byproducts for chickens	10,000	
		Young animals (e.g., piglets)	1000	
		Cattle and poultry feed	5000	
Japan	Food	Wheat	1100	[20]
EU	Food	Unprocessed durum wheat and oats	1750	[21]
		Processed grains (flour)	750	
		Cereals for infants and young children	200	
		Bread, pastries, biscuits, cereal snacks, and breakfast cereals	500	
	Feed	Compound feed for calves, lambs	2000	
		Compound feed	5000	
		Compound feed for pigs	900	
		Feed materials: maize byproducts	12,000	
Canada	Food	Unprocessed wheat, barley, corn	2000	[20]
		Soft wheat flour (baby food)	200	
	Feed	Pigs (full-price feed)	1000	

**Table 3:** Main Raw Material Sources and Contamination Levels of DON.

States	Ingredient	Number of Positives/Samples	Contamination Rate	References
China	Green fodder	199/200	99.5%	[32]
	Whole pig feed	128/129	99.2%	[18]
	Wheat flours	349/359	97.2%	[26]
Germany	Forage corn	120/120	100%	[27]
	White wheat flours	28/28	100%	[33]
	Whole-grain wheat flours	19/19	100%	
USA	Piglet feed	143/144	99.3%	[29]
	Corn silage	1117/1266	88.2%	[30]
	White flours	141/272	51.8%	[34]
	Whole wheat flours	36/90	40.0%	
Poland	Complete feed	2005/2013	99.6%	[28]
Brazil	Barley	72/76	94.7%	[31]
Spain	Cereal for infants and toddlers	12/30	40.0%	[35]

## Data Availability

The original contributions presented in this study are included in the article. Further inquiries can be directed to the corresponding author.
